# Humoral Responses and Chronic GVHD Exacerbation after COVID-19 Vaccination Post Allogeneic Stem Cell Transplantation

**DOI:** 10.3390/vaccines10020330

**Published:** 2022-02-18

**Authors:** Caroline Pabst, Louise Benning, Nora Liebers, Maike Janssen, Leandra Caille, Claudius Speer, Lixiazi He, Maria-Luisa Schubert, Laura Simons, Ute Hegenbart, Stefan Schönland, Aleksandar Radujkovic, Michael Schmitt, Paul Schnitzler, Carsten Müller-Tidow, Sascha Dietrich, Peter Dreger, Thomas Luft

**Affiliations:** 1 Department of Medicine V, University Hospital Heidelberg, 69120 Heidelberg, Germany; nora.liebers@med.uni-heidelberg.de (N.L.); maike.janssen@med.uni-heidelberg.de (M.J.); leandra.caille@med.uni-heidelberg.de (L.C.); lixiazi.he@med.uni-heidelberg.de (L.H.); maria-luisa.schubert@med.uni-heidelberg.de (M.-L.S.); laura.simons@med.uni-heidelberg.de (L.S.); ute.hegenbart@med.uni-heidelberg.de (U.H.); stefan.schoenland@med.uni-heidelberg.de (S.S.); aleksandar.radujkovic@med.uni-heidelberg.de (A.R.); michael.schmitt@med.uni-heidelberg.de (M.S.); carsten.mueller-tidow@med.uni-heidelberg.de (C.M.-T.); sascha.dietrich@med.uni-heidelberg.de (S.D.); peter.dreger@med.uni-heidelberg.de (P.D.); thomas.luft@med.uni-heidelberg.de (T.L.); 2 Department of Nephrology, University of Heidelberg, 69120 Heidelberg, Germany; louise.benning@med.uni-heidelberg.de (L.B.); claudius.speer@med.uni-heidelberg.de (C.S.); 3 Department of Infectious Diseases, Virology, University Hospital, 69120 Heidelberg, Germany; paul.schnitzler@med.uni-heidelberg.de

**Keywords:** allogeneic stem cell transplantation, COVID-19, cGVHD, humoral responses, immunosuppression

## Abstract

The COVID-19 pandemic threatens patients with a compromised immune and endothelial system, including patients who underwent allogeneic stem cell transplantation (alloSCT). Thus, there is an unmet need for optimizing vaccination management in this high-risk cohort. Here, we monitored antibodies against SARS-CoV-2 spike protein (anti-S1) in 167 vaccinated alloSCT patients. Humoral immune responses were detectable in 81% of patients after two vaccinations with either mRNA-, vector-based, or heterologous regimens. Age, B-cell counts, time interval from vaccination, and the type of vaccine determined antibody titres in patients without systemic immunosuppression (sIS). Similar to a healthy control cohort, mRNA vaccine-based regimens induced higher titres than vector-based vaccines. Patients on two or more immunosuppressants rarely developed immunity. In contrast, 62% and 45% of patients without or on only one immunosuppressant, respectively, showed a strong humoral vaccination response (titre > 100). Exacerbation of cGVHD upon vaccination was observed in 6% of all patients and in 22% of patients receiving immunosuppression for cGVHD. cGVHD exacerbation and low antibody titres were both associated with higher angiopoietin-2 (ANG2) serum levels. In conclusion, mRNA-based vaccines elicit strong humoral responses in alloSCT patients in the absence of double sIS. Biomarkers such as ANG2 might help with weighing cGVHD risk versus beneficial responses.

## 1. Introduction

Vaccination against COVID-19 is recommended in alloSCT patients as early as three months post transplantation [1]. Previous studies identified immunosuppressive treatment or ongoing chronic graft-versus-host disease (cGVHD), low B-cell counts, B cell-depleting therapy, anti-thymocyte globulin (ATG) prophylaxis, and time from alloSCT as factors significantly impacting humoral vaccination responses post alloSCT [1,2]. However, most patient cohorts were small (<100 patients), which probably explains why the identified prognostic factors partially differed between the studies. Moreover, only a few studies differentiated between the different vaccine types available and stratified patients based on the number of immunosuppressive agents. In addition, the dilemma of trading cGVHD exacerbation off against a SARS-CoV-2 immune response remains unsolved.

## 2. Materials and Methods

### 2.1. Study Design

We monitored antibodies against SARS-CoV-2 spike protein (anti-S1) and nucleocapsid protein (anti-N) in the serum of 167 patients who had undergone alloSCT at the University Hospital Heidelberg between 1996 and 2021 with post-vaccination serum samples available. All patients were vaccinated more than 100 days post alloSCT. Vaccines included AstraZeneca ChAdOx1 (AZ), BioNTech Pfizer BNT162b2 (BP), and Moderna mRNA-1273 (M). Patients provided written informed consent for sample and data collection and the local ethics board approved the study. Patients with positive anti-SARS-CoV-2-N were excluded from the analysis, as they had likely undergone active SARS-CoV-2 infection. A cohort of 134 healthy controls, which were published previously [3], was used as comparison. 

### 2.2. Neutralizing Capacity of SARS-CoV-2 Antibodies in Surrogate Virus Neutralization Assays

We used a surrogate virus neutralization test (Medac, Wedel, Germany) to detect vaccine-induced neutralizing antibodies as previously described [2]. The absorbance of the final solution was read at 450 nm and the percent (%) inhibition was calculated as follows: Percent signal inhibition = (1 − (OD value of Sample/OD value of Negative Control)) × 100%. With a cutoff of ≥30% inhibition of RBD:ACE-2 binding, the test achieves 99.93% specificity with 95–100% sensitivity to detect surrogate neutralizing antibodies.

### 2.3. Cytokine Levels

Interleukin 18 (IL18, n = 110), interferon gamma (IFNg, n = 108), Angiopoietin-2 (ANG2, n = 103), and CXCL9/MIG (MIG, n = 1 08) were determined if serum was available, including for 21 patients before the first vaccination, using ELISAs (Duo sets, R&D, Abingdon, United Kingdom) as described [4,5,6].

### 2.4. Statistical Analysis

Continuous variables were log10-transformed. The nondimensional anti-S1 index was capped above 150. Therefore, patients were categorized using thresholds as described below. Univariate analyses and visualization were performed in R and GraphPad prism version 9.0.1. Unpaired *t*-tests were used to compare continuous variables. *p*-values were Benjamini–Hochberg (BH) adjusted. Contingency tables were analyzed with Fisher’s exact test. Multivariate and correlation analyses were performed in SPSS version 28.0. *p*-values < 0.05 were considered significant.

## 3. Results

**Figure 1:**
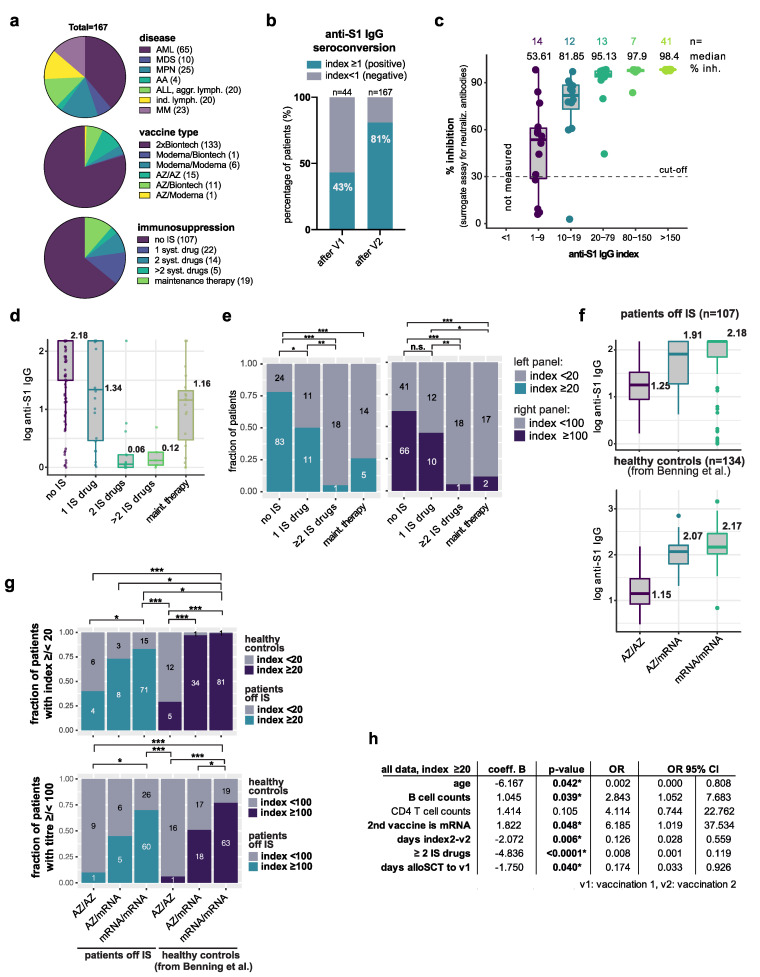
Humoral responses after COVID-19 vaccination in 167 post alloSCT patients. (**a**) Pie charts visualizing the distribution of disease types (above), vaccine types (middle), and number of IS agents employed (below) in the total cohort of 167 patients post alloSCT. Patients on IS (n = 41) were grouped according to the number of systemic drugs applied and comprised cyclosporine A serum level > 50 µg/L, tacrolimus or sirolimus serum level > 3 µg/L, any dose of corticosteroid, mycophenolate mofetil (MMF), or ruxolitinib. Topical treatment and extracorporeal photopheresis were not considered; (**b**) Bar graph displaying the fraction of patients achieving seroconversion defined as anti-S1 IgG index ≥ 1 after the first (n = 44) or second (n = 167) vaccination. v1: first vaccination, v2: second vaccination. (**c**) Percent inhibition determined by a surrogate virus neutralization test to estimate the amount of neutralizing antibodies in sera of patients with anti-S1 IgG ≥ 1. Patients were grouped according to anti-S1 IgG response. In this cohort, all patients with an anti-S1 IgG > 20 reached the cutoff of 30% inhibition (see methods for details). Numbers on the top indicate the numbers of patients in each group. Median percent inhibition is indicated in the second row; (**d**) Box plot showing log-transformed anti-S1 IgG titres according to the number of immunosuppressive drugs employed. Horizontal lines and corresponding numbers indicate the median. Dots represent individual patient samples. Maint. Therapy: maintenance therapy; (**e**) Fraction of patients with index </≥ 20 (left panel) and </≥ 100 (right panel) according to the number of sIS drugs employed. Numbers in bars indicate the numbers of patients in each group. Pairwise Fisher’s exact tests, BH-adjusted, * *p* < 0.05, ** *p* < 0.005, *** *p* < 0.0005, n.s. not significant; (**f**) Box plot showing log-transformed anti-S1 IgG index values in patients off IS (above) or in healthy controls. Horizontal lines and numbers indicate the median, points indicate outliers (Tukey method); (**g**) Fraction of patients with index </≥ 20 (above) and </≥ 100 (below) according to the vaccination regimen employed. Left three bars show patients off IS, while the right three bars show healthy controls. AZ/AZ: homologous vaccination with AZ, AZ/mRNA: heterologous regimen with AZ followed by either BP or M, mRNA/mRNA: homologous vaccination with either BP or M as first and second vaccine. See Table A1 for further details. Numbers in bars indicate the numbers of patients in each group. Pairwise Fisher’s exact tests, BH-adjusted, * *p* < 0.05, ** *p* < 0.005, *** *p* < 0.0005, n.s. not significant; (**h**) Logistic regression identifies age, number of IS drugs, B cell counts, type of vaccine, and interval from vaccination as independent factors associated with humoral response post COVID-19 vaccination in the total cohort of 167 patients post alloSCT. OR: odds ratio, * *p* < 0.05.

In the total cohort of 167 patients, 81% exhibited a positive immune response after two vaccinations (detailed patient characteristics are shown in Figure 1a and Table A1, anti-S1 index ≥ 1, Figure 1b). Among the 44 patients with serum samples available after both vaccination time points, 43% showed seroconversion already after the first dose (Figure 1b). Given that the anti-S1 index does not identify neutralizing antibodies, we applied a surrogate virus neutralization test to detect vaccine-induced neutralizing antibodies in the sera of patients with an anti-S1 index ≥ 1. Not all patients reached the threshold of 30% inhibition in the surrogate assay, when the index was <20 (Figure 1c). In contrast, all sera from patients with an index ≥ 20 showed inhibition beyond the cut-off of 30%, with a median inhibition > 95% (Figure 1c). We, therefore, included a threshold of ≥20 in further analyses to compare patient groups. 

**Figure A1:**
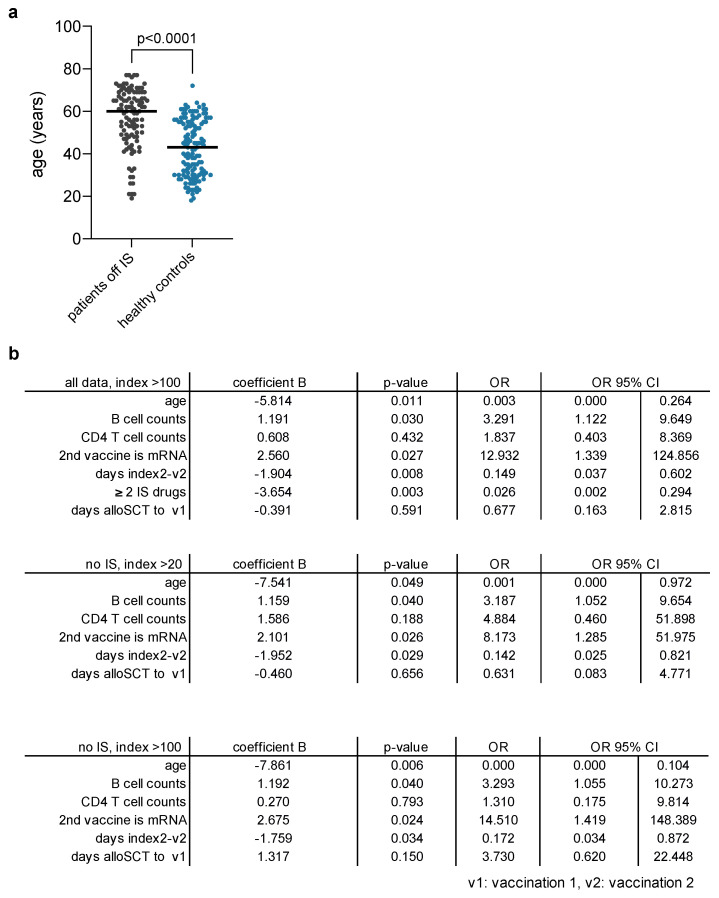
Factors impacting vaccination response in alloSCT patients undergoing COVID-19 vaccination. (**a**) Age (years) of patients off IS versus healthy controls. Lines indicate medians. Mann Whitney U test; (**b**) Logistic regression identifies independent factors associated with humoral responses post COVID-19 vaccination in the total cohort (index > 100, top), and in the subgroup of patients off IS (n = 107, index > 20, middle, index > 100, bottom). v1: first vaccination, v2: second vaccination, continuous variables were log10-transformed, OR odds ratio.

The anti-S1 index significantly decreased with increasing numbers of sIS drugs used (Figure 1d). While 10 of 22 patients treated with only one systemic immunosuppressant achieved a high index > 100, 19 of 20 patients on two or more systemic IS drugs did not reach an index > 20 (Figure 1e). The vaccination response of patients receiving maintenance therapies (e.g., ´IMiDs or kinase inhibitors) was also significantly lower compared to patients off IS (Figure 1e). We next analyzed the 107 patients off IS separately and identified the vaccine regimen as an important response-determining factor. The median (IQR) titre was 16 (0.18 to >150) with homologous AZ vaccination versus 81 (3.23 to >150) and 150 (0 to >150) with heterologous or homologous mRNA vaccine-based strategies, respectively (Figure 1f). While healthy controls receiving mRNA vaccines performed slightly better than patients off IS at threshold 20, there was no difference in IgG responses between healthy individuals and patients post SCT off IS when using a cutoff > 100 (Figure 1g). These concordant results were specifically striking, as patients off IS were about 20 years older than healthy controls (*p* < 0.0001, Figure A1a). In a multivariate logistic regression analysis, treatment with more than one sIS drug was the most significant independent predictor of poor vaccination responses, followed by higher age, low B-cell counts, AZ vaccine only, and the interval from second vaccination (Figure 1h, Figure A1b). When analyzing patients based off IS separately, age, vaccine type and time interval remained independent determinants of the anti-S1 index, with both thresholds (>20; >100) (Figure A1b). 

**Figure 2:**
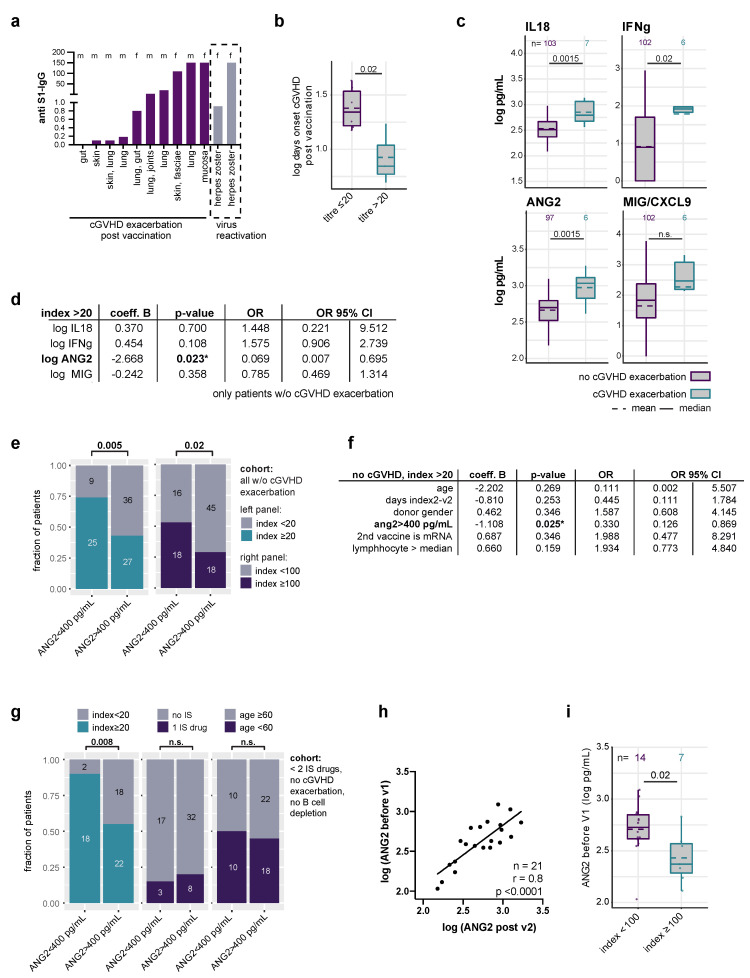
Adverse events and biomarkers in alloSCT patients undergoing COVID-19 vaccination. (**a**) Bar graph indicating the anti-S1 IgG level of 10 patients suffering exacerbation of cGVHD post vaccination and 2 patients with herpes zoster post vaccination. Exacerbation of cGVHD was defined as the need for IS treatment escalation or re-start of IS treatment in patients off IS at the time of the first vaccination. Letters on top indicate patients’ gender showing no enrichment of either gender; organs below bars indicate the organs affected by cGVHD; (**b**) Boxplot indicating the mean (dashed line) and median (horizontal line) log days between vaccination and onset of symptoms in patients with index > 20. Unpaired *t*-test, *p* = 0.02; (**c**) Log-transformed cytokine levels (pg/mL) of four cytokines indicated on the top of each plot in patients suffering exacerbation or aggravation of cGVHD (petrol) versus all others (violet). Unpaired *t*-test. Dashed line: mean, horizontal line: median; (**d**) logistic regression identifies ANG2 levels as the only one of the four cytokines significantly associated with an index > 20. Patients with exacerbation of cGVHD were excluded from this analysis. OR: odds ratio; (**e**) Fraction of patients with an index > 20 (left) or > 100 (right) when ANG2 levels were below or above 400 pg/mL. Fisher’s exact test; (**f**) Logistic regression identifies ANG2 as an independent factor associated with an index > 20 in the total cohort excluding patients suffering cGVHD exacerbation. v2: second vaccination, * *p* < 0.05; (**g**) Left: fraction of patients with index > 20 with high versus low ANG2 levels. Middle and right panel: there was no significant difference in the fraction of patients with one sIS drug or age > 60 years in the two ANG2 groups. Fisher´s exact test; (**h**) Pearson correlation between ANG2 levels before the first and after the second vaccination. Values (pg/mL) were log-transformed; (**i**) Log-transformed ANG2 levels in patients before first vaccination who achieved titres > 100 after the second vaccination. Dashed line and line indicate means and medians. Points represent individual patient samples. Numbers on top indicate numbers of patients in each group.

**Figure A2:**
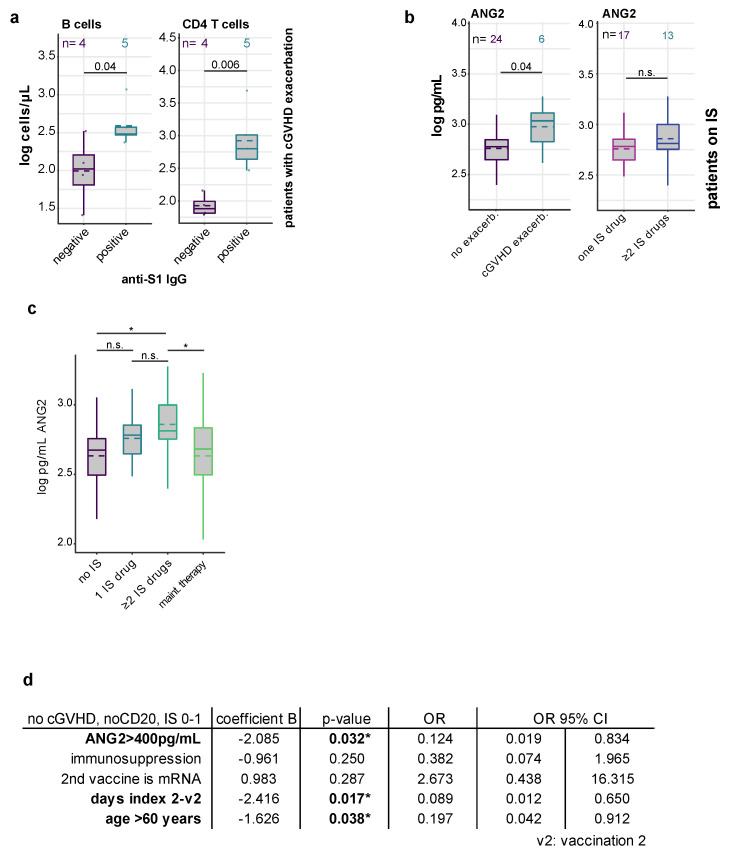
Factors associated with vaccination response and adverse events in alloSCT patients following COVID-19 vaccination. (**a**) Box plots showing CD4 and B cell counts (log cells/µL) in patients with cGVHD exacerbation according to positive or negative IgG test (anti-S1 IgG </> 1). Mean (dashed line) and median (horizontal line), points represent individual samples, numbers on the top indicate sample size per group, unpaired *t*-test; (**b**) Log10-transformed ANG2 levels (pg/mL) in patients on IS treatment suffering exacerbation of cGVHD (left panel, petrol) versus all others (violet) or in patients with one or more immunosuppressive drugs (right panel). Unpaired *t*-test. Dashed line: mean, horizontal line: median; (**c**) Log10-transformed ANG2 levels (pg/mL) according to sIS treatment. Note that patients on two or more sIS drugs have significantly higher ANG2 levels and were therefore excluded from the subsequent logistic regression analysis; (**d**) Logistic regression analysis reveals high ANG2 as an independent factor associated with an index > 20 in the subgroup of patients with less than two sIS drugs, no B cell depleting therapy and no cGVHD exacerbation, as these conditions might influence ANG2 serum levels.* *p* < 0.05.

Potential immunological risks of COVID-19 vaccination were identified in 10 individuals (5,9%), who exhibited an exacerbation of cGVHD within 80 days following vaccination (median 17 days, range 7–74 days). Exacerbation or aggravation of cGVHD was defined as the requirement for IS treatment escalation or re-start of IS treatment in patients off IS following COVID-19 vaccination. Nine of these ten patients were on sIS treatment for smoldering or on tapering sIS for controlled cGVHD (9 of 41, 22%). All patients received systemic corticosteroids; some needed additional agents, including calcineurin inhibitors, MMF, and topical treatment. Two patients suffered another cGVHD exacerbation after the third vaccination. cGVHD exacerbation events seemed to be independent of the index (Figure A2a). Two patients (1%) developed herpes zoster at 7 and 14 days post vaccination, which is in the typical range described for herpes zoster after COVID-19 infection or mRNA vaccination [7,8] (Figure 2a). Regarding cGVHD aggravation, we observed an association between a stronger vaccination response and shorter time to onset of symptoms (Figure 2b). To corroborate that the identified patients suffered cGVHD, we measured four cytokines that were previously found to be associated with GVHD and/or poor outcome post alloSCT (IL18, ANG2, CXCL9/MIG, IFNg) [4,5,6,9]. Concordantly, cytokine levels were significantly higher (Il18, ANG2, IFNg) in patients with suspected cGVHD aggravation (Figure 2c). For ANG2, this difference was also detectable when considering only patients on IS (Figure A2b). We next asked whether any of these cytokines correlated with the anti-S1 IgG index and found a significant association only for ANG2 (Figure 2d–f, Figure A2c). 

Moreover, patients on two or more sIS drugs showed significantly higher ANG2 levels than the others (Figure A2c). When excluding patients with cGVHD exacerbation, two or more sIS drugs or history of anti-CD20 therapies, ANG2 remained an independent factor associated with a poorer response (Figure 2g, Figure A2d). In 21 patients who showed serum available from a maximum of 30 days prior to first vaccination, a strong correlation of serum ANG2 levels before and after vaccination was observed (Figure 2h). Accordingly, patients with index > 100 had lower ANG2 levels already before the first vaccination compared with the others (Figure 2i). 

## 4. Discussion

We described the humoral responses after COVID-19 vaccination in a large cohort of 167 post alloSCT patients. Strong anti-S1 humoral immune responses are observed with current SARS-CoV-2 vaccines in the majority of alloSCT recipients off immunosuppression eliciting titres similar to those detected in healthy controls [3]. In contrast to other studies with smaller patient cohorts, our study comprised both mRNA- and vector-based vaccines [1,10]. It clearly shows that tandem mRNA-based vaccinations or combination strategies consisting of vector first followed by mRNA appear to elicit considerably higher titres than tandem vector vaccinations. While all studies identify immunosuppression as a major factor negatively impacting vaccination response [1,2,10], we stratified the patients according to the type, serum levels, and combinations of immunosuppressants and found that about half of the patients on only one immunosuppressant were still able to develop high IgG titres. Exacerbation of cGVHD upon vaccination occurred in only 6% of all patients, as reported before [10]. However, this number rises to 22% of patients receiving immunosuppression for cGVHD and should, therefore, be considered in the vaccination management of patients with smouldering or with a recent history of severe cGVHD. As most patients in our cohort were in complete remission with full donor chimerism, we received no indication as to whether COVID-19 vaccination, besides inducing cGVHD in some patients, supports graft-versus-tumor (GVT) effects. While exacerbation of cGVHD post vaccination was being controlled in all cases, it often required application of high-dose steroids besides dose escalation of the ongoing sIS treatment. Dose escalation of sIS not only hampers sustained anti-S1 IgG titres, but also bears the risk of infections and relapse. The search for biomarkers to help decide whether to vaccinate patients with cGVHD against SARS-CoV-2 is, therefore, warranted. High ANG2 levels had previously been associated with endothelial damage and had, therefore, been shown to predict poor outcome in patients suffering active SARS-CoV-2 infection [11]. We described here that high ANG2 levels were also associated with lower titres post vaccination in particular in patients off IS. Whether ANG2 may serve as a biomarker prior to vaccination to estimate the chances of successful vaccination versus cGVHD aggravation and whether there is a mechanistic link between high ANG2 serum levels and low humoral responses will have to be addressed in future studies.

## 5. Conclusions

In conclusion, mRNA should be preferred to vector-based vaccines in alloSCT recipients. Double immunosuppression should be avoided, and the risk of cGVHD exacerbation must be considered in the vaccination strategy. Further studies are needed to explore biomarkers that may help weighing cGVHD risk versus immune response. 

## Data Availability

Not applicable.

## Appendix A

**Table A1:** Patient characteristics.

	Values	Median	Percentage (%)
**Total Patients post alloSCT**	167		
disease type			
AML	65		38.9
MDS	10		6.0
MPN	25		15.0
Aplastic Anemia	4		2.4
ALL, aggressive lymphoma	20		12.0
indolent lymphoma	20		12.0
Multiple Myeloma	23		13.8
age	19–79	60	
age above 60 years	80		47.9
sex			
female	65		38.9
male	102		61.1
Days last alloSCT to 1st vaccination			
	106–9111	1215	
Remission status			
complete remission	158		94.6
Immunosuppression (IS)			
no IS	107		64.1
one systemic IS drug	22		13.2
2 systemic IS drugs	14		8.4
3 or more systemic IS drugs	5		3.0
total IS	41		24.6
maintenance therapy	19		11.4
Seroconversion			
positive S-AK after V1	19/44		43.2
positive S-AK after V2	135/167		80.8
positive N-AK after V2	0		0.0
vaccines			
AZ/AZ	15		9.0
AZ/Moderna	1		0.6
AZ/Biontech	11		6.6
Biontech/Biontech	133		79.6
Moderna/Biontech	1		0.6
Moderna/Moderna	6		3.6
type of regimen			
homologous AZ/AZ	15		9.0
heterolohous AZ/mRNA	12		7.2
homologous mRNA/mRNA	140		83.8

V1: vaccination 1, V2: vaccinaton 2, AZ Astra Zeneca.
